# Beneficial Effects of Wheat Gluten Hydrolysate to Extend Lifespan and Induce Stress Resistance in Nematode *Caenorhabditis elegans*

**DOI:** 10.1371/journal.pone.0074553

**Published:** 2013-09-09

**Authors:** Weiming Zhang, Ting Lv, Min Li, Qiuli Wu, Linsong Yang, Hui Liu, Dafeng Sun, Lingmei Sun, Ziheng Zhuang, Dayong Wang

**Affiliations:** 1 College of Life Sciences, Nanjing Normal University, Nanjing, China; 2 Nanjing Institute for Comprehensive Utilization of Wild Plants, Nanjing, China; 3 School of Pharmaceutical Engineering and Life Sciences, Changzhou University, Changzhou, China; 4 Key Laboratory of Developmental Genes and Human Disease in Ministry of Education, Medical School of Southeast University, Nanjing, China; University of Houston, United States of America

## Abstract

Previous studies have showed that wheat gluten hydrolysate (WGH) has the anti-oxidative property. In the present study, we examined the possible safety property of WGH and the beneficial effects of WGH to extend lifespan and induce stress resistance using nematode *Caenorhabditis elegans* as the *in vivo* assay system. We found that WGH at concentrations of 0.1–1 mg/mL did not cause lethality, influence development, alter locomotion behavior and brood size, and induce significant intestinal autofluorescence and reactive oxygen species (ROS) production in young adults. Treatment with 0.1–1 mg/mL of WGH significantly extended lifespans of nematodes under the normal conditions. Moreover, WGH treatment significantly inhibited the induction of intestinal autofluorescence and suppressed the decrease in locomotion behavior during the aging process of nematodes. Furthermore, pre-treatment with 1 mg/mL of WGH significantly suppressed the adverse effects caused by heat-stress or oxidative stress on nematodes as indicated by the alterations of both lifespan and intestinal ROS production. Therefore, WGH treatment is relatively safe and has beneficial effects on nematodes under both the normal conditions and the stress conditions.

## Introduction

Wheat, one of the most important grain crops, is a staple food for more than 30% population worldwide and an important source of industry products. Wheat gluten, an important byproduct of wheat starch industry, is recognized as a source of dietary protein [1]. However, utilization of wheat gluten as a food is limited during application because it may induce some severe diseases such as celiac disease [2]. In contrast, enzymatic hydrolysates of wheat gluten (wheat gluten hydrolysate, WGH) might be safe [3], and have been demonstrated to have *in vitro* antioxidant activities [4]. Moreover, previous studies have implied that WGH may have some beneficial effects on human beings, for example post-training consumption of WGH suppressed the delayed onset of muscle injury in well-trained college runners and soccer players [5]–[6]. Just recently, model animals such as rat were further used to investigate the benefit effects of WGH against D-galactosamine-induced acute hepatitis [7]. The anti-oxidative property of WGH implies that WGH can serve as an anticipated functional food or dietary supplement. Nevertheless, the *in vivo* evidence is still largely limited to support this assumption.

Nematode *Caenorhabditis elegans* is a model animal widely used in biomedical and toxicological research and acts as a non-mammalian alternative toxicity assay model [8]–[10]. *C. elegans* can be used for safety assessment of many toxicants such as heavy metal, drug, and nanomaterials [11]–[14]. The facts that genome and important signal pathways are conserved between *C. elegans* and human and aging process of *C. elegans* is similar to human make it an ideal model for aging research and anti-aging drug discovery [15]–[16]. Meanwhile, *C. elegans* can also be used to screen and examine the beneficial effects of specific drugs with properties against oxidative stress or extending lifespan [17]–[23]. In the present study, we examined the possible safety property of WGH using *C. elegans* as the *in vivo* assay system. Moreover, we investigated whether WGH has anti-aging or anti-stress effects on animals.

## Results

### Safety evaluation of WGH on *C. elegans*

**Figure 1:**
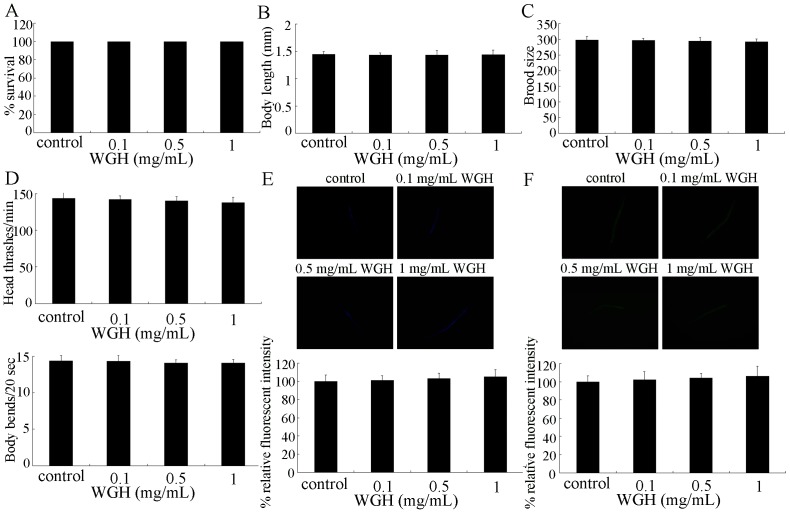
Toxicity assessment of WGH on *C. elegans*. (A) Effects of WGH on lethality. (B) Effects of WGH on growth. (C) Effects of WGH on reproduction. (D) Effects of WGH on locomotion behavior as indicated by head thrash and body bend. (E) Effects of WGH on intestinal autofluorescence. (F) Effects of WGH on intestinal ROS production. WGH treatment was performed for 24-hr from L4-larvae. WGH, wheat gluten hydrolysate. Bars represent means ± S.E.M.

After acute exposure from L4-larvae for 24-hr, we assessed the possible safety property of WGH using the *in vivo* system of *C. elegans*. Firstly, we found that treatment with 0.1–1 mg/mL of WGH did not induce lethality and influence development of nematodes (Fig. 1A and 1B). Reproductive organs and neurons are secondary targeted organs for drugs or toxicants in *C. elegans* [9], [24]–[25]. Treatment with 0.1–1 mg/mL of WGH did not significantly alter brood size and locomotion behavior of nematodes (Fig. 1C and 1D), suggesting the normal functions of possible secondary targeted organs in WGH treated nematodes. Intestine is the primary targeted organ for drugs or toxicants in *C. elegans* [9], [25]–[26]. Intestinal autofluorescence reflects the accumulation of lipofuscin, and serves as an useful biomarker of the physiological age in *C. elegans* [27]. Moreover, we did not observe noticeable induction of intestinal autofluorescence or intestinal ROS production in 0.1–1 mg/mL of WGH treated nematodes compared with control (Fig. 1E and 1F), implying the normal physiological state of primary targeted organs in WGH treated nematodes.

### WGH treatment extends the lifespan of *C. elegans*

**Figure 2:**
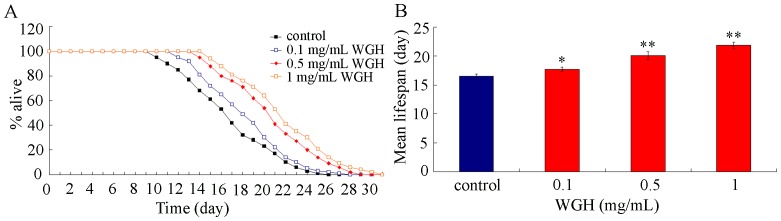
Effects of WGH treatment on lifespan of *C. elegan*. (A) Lifespan curves of nematodes treated with WGH. (B) Comparison of mean lifespans in nematodes treated with WGH. WGH treatment was performed throughout the lifespan of nematodes from L4-larvae. WGH, wheat gluten hydrolysate. Bars represent means ± S.E.M. ^*^ *p*<0.05, ^**^ *p*<0.01.

We next determined the effects of WGH treatment at different concentrations on lifespan in nematodes. As shown in Fig. 2, treatment with the three examined concentrations of WGH all significantly extended lifespans of nematodes. These data demonstrated the potential anti-aging property of WGH as a functional food or dietary supplement.

### WGH treatment decreases intestinal autofluoresence during the aging process

**Figure 3:**
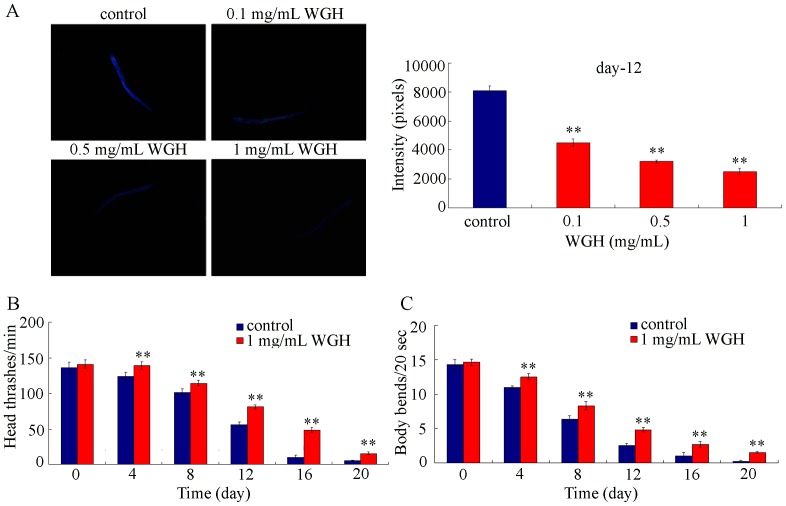
Effects of WGH treatment to decrease intestinal autofluorescence (A) and enhance locomotion behavior (B-C) during the aging process. WGH treatment was performed throughout the lifespan of nematodes from L4-larvae. WGH, wheat gluten hydrolysate. Bars represent means ± S.E.M. ^**^ *p*<0.01.

For wild-type N2 nematodes, the population of dead animals and the accumulation of intestinal autofluorescence increase sharply after adult day-10 [28]. Under our experimental conditions, we observed the significant increase of intestinal autofluorescence at the stage of adult day-12 in control nematodes (Fig. 3A). Compared with the increased intestinal autofluorescence phenotype in control nematodes at the stage of adult day-12, treatment with the three examined concentrations of WGH all significantly suppressed the intestinal autofluorescence of nematodes (Fig. 3A), suggesting the possible amelioration of functions at the primary targeted organs during the aging process in WGH treated nematodes.

### WGH treatment enhances locomotion behavior during the aging process

For wild-type N2 nematodes, the ability of locomotion behavior will be gradually decreased during the aging process [29]. After WGH treatment, locomotion behaviors of treated nematodes were monitored every 4-day during their lifespan. As shown in Fig. 3B and 3C, locomotion behaviors as reflected by endpoints of head thrash and body bend in WGH treated nematodes were significantly enhanced compared with those in control nematodes. These data imply the possibility that WGH treatment may increase the quality of lifespan at least partially by increasing the ability of locomotion behavior during the aging process in nematodes. Therefore, besides the lifespan-extending effect, WGH treatment may also have the beneficial effects on quality of lifespan in *C. elegans*.

### Stress resistance property of WGH in *C. elegans*

**Figure 4:**
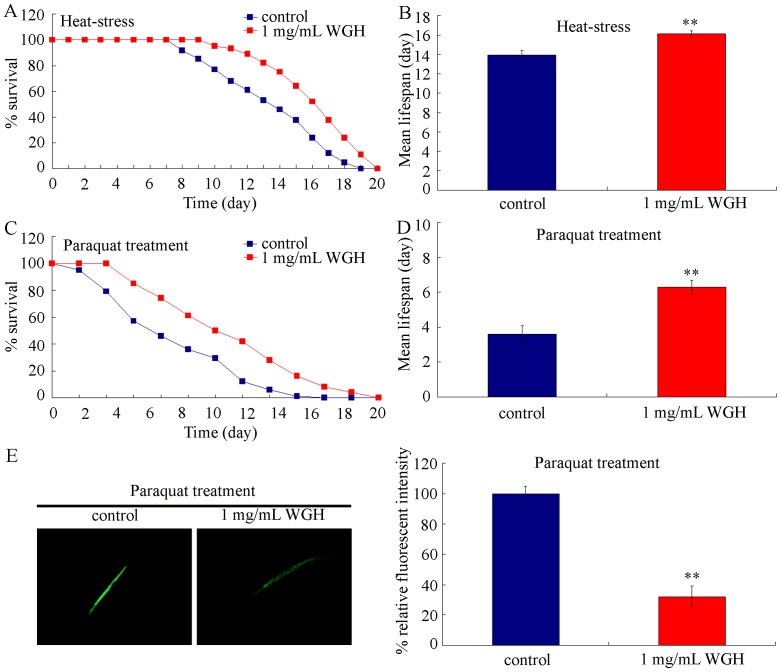
Heat-stress and oxidative stress resistance of WGH treated *C. elegans*. (A) Lifespans of heat-stress exposed nematodes pretreated with or without 1 mg/mL of WGH. (B) Mean lifespans of heat-stress exposed nematodes pretreated with or without 1 mg/mL of WGH. (C) Lifespans of paraquat exposed nematodes pretreated with or without 1 mg/mL of WGH. (D) Mean lifespans of paraquat exposed nematodes pretreated with or without 1 mg/mL of WGH. (E) Intestinal ROS production in paraquat exposed nematodes pretreated with or without 1 mg/mL of WGH. Nematodes pre-treated with 1 mg/mL of WGH for 48-hr were transferred to the 35°C condition for 16–hr or 2 mmol/L of paraquat for 6–hr. WGH, wheat gluten hydrolysate. Bars represent means ± S.E.M. ^**^ *p*<0.01.

Previous studies showed that some drugs having lifespan-extending property can also increase lifespan of *C. elegans* under hostile conditions such as heat-stress or oxidative stress [21], [30]. To investigate whether WGH has stress resistance property, nematodes pretreated with 1 mg/mL of WGH for 48-hr were further exposed to heat-stress (35°C) for 16-hr or 2 mmol/L of paraquat, a ROS-generator, for 6–hr. After 1 mg/mL of WGH treatment, nematodes exhibited a significant resistance to both heat-stress and oxidative stress (Fig 4A–4D). Moreover, we found that pretreatment with 1 mg/mL of WGH significantly inhibited the induction of intestinal ROS production induced by 2 mmol/L of paraquat in nematodes (Fig. 4E).

## Discussion

In the present study, we first provided the evidence to demonstrate the possible safety profiles of WGH with the aid of *in vivo* assay system of *C. elegans*. In the examined concentrations, WGH treatment did not induce lethality and influence development of nematodes (Fig. 1A and 1B). Moreover, WGH treatment at the examined concentrations did not alter locomotion behavior and brood size (Fig. 1C and 1D), and induce the significant intestinal autofluorescence and ROS production (Fig. 1D and 1E), suggesting the normal physiological state of possible primary and secondary targeted organs for WGH in nematodes. Therefore, our *in vivo* data demonstrated that WGH had not induced adverse effects on the examined endpoints in wild-type animals.

Previous studies have showed that WGH has anti-oxidant ability [4]–[6], which makes it interest to investigate whether WGH will have beneficial effects, such as lifespan-extension, on animals. Our data here demonstrated that WGH treatment at the examined concentrations significantly extended the lifespans of nematodes under the normal conditions (Fig. 2). Moreover, WGH treatment significantly inhibited the induction of intestinal autofluorescence and suppressed the decrease in locomotion behavior during the aging process of nematodes (Fig. 3). These results suggest that WGH not only extends the lifespan but also improves the quality of life by modulating the age-associated alterations in *C. elegans.* Our data also imply that WGH may have multiple beneficial effects as a functional food or dietary supplement, which is largely consistent with the previous studies [4]–[7].

Moreover, our data demonstrated that under both heat-stress and oxidative stress conditions, pre-treatment with 1 mg/mL of WGH significantly suppressed the adverse effects of heat-stress and oxidative stress on nematodes as indicated by alterations of both lifespan and ROS production (Fig. 4). These data imply that the beneficial effects of WGH can be observed not only under the normal conditions but also under the stress conditions. However, our data indicated that WGH treatment could not completely recover the deficits of nematodes induced by heat-stress and oxidative stress, which suggests that WGH can not act as an alternative for clinical drug with the detoxification function.

In *C. elegans*, low concentrations of juglone, another ROS-generator, induced the adaptive response and caused a prolongation of lifespan; however, high concentrations of juglone did not induce the adaptive response but led to the premature death [31]–[32]. Similarly, pre-treatment with mild metal exposure or UV-irradiation induced the hormesis or cross-adaptation; however, pre-treatment with severe metal exposure or UV-irradiation would not further induce the hormesis or cross-adaptation in *C. elegans* [10], [33]–[35]. The used concentration of paraquat (2 mmol/L) in the current study was relatively high and would not induce the adaptive response. However, it was interestingly found that WGH treatment could prolong the lifespan of nematodes and largely inhibit the toxicity induced by paraquat (Figs. 1 and 4). Previous studies further demonstrated that administration with ascorbic acid, a hydrophilic antioxidant or ROS-scavenger, prolonged the lifespan of nematodes [36], and blocked the lifespan reduction induced by high concentrations of juglone in *C. elegans* [32]. Therefore, although we still can not exclude the other possibilities, WGH treatment may induce an adaptive response by acting as a ROS-scavenger to extend the lifespan and suppress the adverse effects from heat-stress and oxidative stress in *C. elegans*.

In conclusion, in the current study we provided the systematic *in vivo* evidence to suggest the relatively safe property of WGH. Our data also indicated the beneficial effects of WGH for nematodes under both the normal conditions and the stress conditions. However, we still do not know whether WGH treatment will be safe for other animals or human. In addition, identification and clarification of specific fractions in WGH with beneficial effects will be helpful for our further understanding the beneficial functions of WGH as a functional food or dietary supplement.

## Materials and Methods

### Reagents and *C. elegans* strain preparation

WGH is a commercial product from Nisshin Pharma (Tokyo, Japan). WGH contained 2.9% water, 77.5% protein, 0.22% natrium, and 0.2% lipid. The amino acid composition of used WGH was summarized in Table S1. All the other chemicals were obtained from Sigma-Aldrich (St. Louis, MO, USA).

Wild-type *C. elegans* N2, originally obtained from *Caenorhabditis* Genetics Center, was maintained on nematode growth medium (NGM) plates seeded with *Escherichia* OP50 at 20°C as described [37]. Age synchronous populations of L4-larvae nematodes were obtained as described previously [38]. Stock WGH solution (500 mg/mL) was prepared in water. WGH was added to the NGM plates to a final concentration of 0.1–1 mg/mL just before plating. Because we would treat the nematodes with WGH throughout the lifespan from the stage of L4-larvae, we here selected this exposure route. Although we can not guarantee the adequate uptake of WGH by nematodes, the previous study has demonstrated that this exposure route for drug administration was effective in nematodes [23]. Gentamycin (30 µg/mL) was added to the NGM plates to inhibit microbial contamination. *E. coli* OP50 was spread on the NGM plates as the food for nematodes. For the safety assessment, lethality, growth, brood size, locomotion behavior, intestinal autofluorescence, and intestinal reactive oxygen species (ROS) production were used as endpoints.

### Lethality, growth, reproduction, and locomotion behavior

Methods were performed as described previously [13]. WGH treatment was performed for 24-hr from the stage of L4-larvae for lethality, growth, and reproduction assay. For lethality assay, nematodes were judged to be dead if they did not respond to stimulus using a small, metal wire. Approximately 50 nematodes were used for each assay. Growth was assessed by body length, which was determined by measuring flat surface area of nematodes using Image-Pro® Express software. Reproduction was assessed by the brood size, which was determined as the number of offspring at all stages beyond the egg was counted. Ten replicates were performed.

For assay of locomotion behavior (head thrash and body bend), WGH treatment was performed for 24-hr or throughout the lifespan from the stage of L4-larvae. To assay locomotion behavior, the examined nematodes were washed with K medium, and transferred into a microtiter well containing 60 µL of K medium on the top of agar. After a 1-min recovery period, head thrashes, defined as a change in the direction of bending at the mid body, were counted for 1-min. Body bends, defined as a change in the direction of the part of nematodes corresponding to the posterior bulb of the pharynx along the *y* axis, assuming that nematode was traveling along the *x* axis, were counted for 20-sec. Twenty replicates were performed.

### Intestinal autofluorescence and ROS production

Methods were performed as described previously [13]. WGH treatment was performed for 24-hr or throughout the lifespan from the stage of L4-larvae. Intestinal autofluorescence caused by lysosomal deposits of lipofuscin can accumulate over time in aging nematodes [39]. For intestinal autofluorescence assay, images were collected for fluorescence in endogenous intestine using a 525-nm bandpass filter and without automatic gain control to preserve relative intensity of fluorescence in animals. Fluorescence was recorded and color images were taken for documentation of results with Magnafire® software (Olympus, Irving, TX, USA). Lipofuscin levels were measured using ImageJ Software (NIH Image, Bethesda, MD, USA) by determining mean pixel intensity in intestines. To analyze ROS production, the examined nematodes were transferred to 1 mL of M9 buffer containing 1 µM CM-H2DCFDA in 12-well sterile tissue culture plates to pre-incubate for 3-hr at 20°C, and then mounted on 2% agar pads for examination with a laser scanning confocal microscope (Leica, TCS SP2, Bensheim, Germany) at 488 nm of excitation wavelength and 510 nm of emission filter. Relative fluorescent intensities of intestines were semi-quantified, and semiquantified ROS was expressed as relative fluorescent units (RFU). Twenty replicates were examined.

### Lifespan assay

Lifespan assay was performed basically as described [15], [40]. WGH treatment was performed throughout the lifespan from the stage of L4-larvae. In this test, the hermaphrodites were transferred daily for the first 4 days of adulthood. Nematodes were checked every 2 days and would be scored as dead when they did not move even after repeated taps with a pick. For lifespan, graphs are representative of at least three trials.

### Heat stress and oxidative stress resistance assays

Nematodes pre-treated with 1 mg/mL of WGH for 48-hr were transferred to the 35°C condition for 16–hr or 2 mmol/L of paraquat for 6–hr [30], [41], and then the lifespan and the intestinal ROS production were measured as described above. Paraquat was used to induce the oxidative stress in nematodes [30].

### Amino acid analysis

Amino acid analysis was performed by the method described previously by Higaki-Sato et al. [42].

### Statistical analysis

All data in this article were expressed as means ± standard error of the mean (S.E.M.). Graphs were generated using Microsoft Excel (Microsoft Corp., Redmond, WA). Statistical analysis was performed using SPSS 12.0 (SPSS Inc., Chicago, USA). Differences between groups were determined using analysis of variance (ANOVA). Probability levels of 0.05 and 0.01 were considered statistically significant. The lifespan data were statistically analyzed using a 2-tailed 2 sample *t*-test (Minitab Ltd., Coventry, UK).

## Supporting Information

Table S1 **Amino acid composition of used WGH.**
(DOC)
