# Supplementary material for: Beneficial Effects of Wheat Gluten Hydrolysate to Extend Lifespan and Induce Stress Resistance in Nematode Caenorhabditis elegans
Source: PLoS One. 2013 Sep 9;8(9):e74553. doi: 10.1371/journal.pone.0074553 (PMC3767650; doi:10.1371/journal.pone.0074553)
Supplement: Table S1 — Amino acid composition of used WGH. (DOC) [file pone.0074553.s001.doc]

**Supplementary Information:**

Table S1. Amino acid composition of used WGH

| Amino acid | Content (%) |
| --- | --- |
| Glycine | 2.946 |
| Alanine | 2.342 |
| Valine | 3.267 |
| Leucine | 4.722 |
| Isoleucine | 2.465 |
| Proline | 8.669 |
| Phenylalanine | 3.976 |
| Lysine | 1.153 |
| Histidine | 1.098 |
| Tyrosine | 1.394 |
| Serine | 3.387 |
| Threonine | 1.148 |
| Cysteine | 0.164 |
| Methionine | 0.113 |
| Aspartic acid | 2.355 |
| Glutamic acid | 30.573 |
| Arginine | 1.792 |
